# Actigraph assessment for measuring upper limb activity in unilateral cerebral palsy

**DOI:** 10.1186/s12984-019-0499-7

**Published:** 2019-02-22

**Authors:** Elena Beani, Martina Maselli, Elisa Sicola, Silvia Perazza, Francesca Cecchi, Paolo Dario, Irene Braito, Roslyn Boyd, Giovanni Cioni, Giuseppina Sgandurra

**Affiliations:** 1 Department of Developmental Neuroscience, IRCCS Fondazione Stella Maris, Viale del Tirreno 331, 56128 Calambrone, Pisa, Italy; 2 The BioRobotics Institute, Scuola Superiore Sant’ Anna, Viale Rinaldo Piaggio 34, 56025 Pontedera, Pisa, Italy; 3 Queensland Cerebral Palsy and Rehabilitation Research Centre, School of Medicine, The University of Queensland, Brisbane, Australia; 4 Department of Clinical and Experimental Medicine, University of Pisa, Via Roma, 56125 Pisa, Italy

**Keywords:** Congenital hemiplegia, Arm movements, Actigraphy, Assisting hand assessment, Information and communications technology (ICT)

## Abstract

**Background:**

Detecting differences in upper limb use in children with unilateral cerebral palsy (UCP) is challenging and highly dependent on examiner experience. The recent introduction of technologies in the clinical environment, and in particular the use of wearable sensors, can provide quantitative measurement to overcome this issue.

This study aims to evaluate ActiGraph GT3X+ as a tool for measuring asymmetry in the use of the two upper limbs (ULs) during the assessment with a standardized clinical tool, the Assisting Hand Assessment (AHA) in UCP patients aged 3–25 years compared to age-matched typically developing (TD) subjects.

**Methods:**

Fifty children with UCP and 50 TD subjects were assessed with AHA while wearing ActiGraphs GT3X+ on both wrists. The mean activity of each hand (dominant and non-dominant, MA_DH_ and MA_NDH_, respectively) and the asymmetry index (AI) were calculated. Two linear mixed model analyses were carried out to evaluate how dependent actigraphic variables (i.e. MA_NDH_ and AI) varied by group (TD vs UCP) and among levels of manual ability based on Manual Ability Classification System (MACS). In both models age, sex, side of hemiplegia, presence/absence of mirror movements were specified as random effects.

**Results:**

The MA_NDH_ was significantly lower in UCP compared to TD, while the AI was significantly higher in UCP compared to TD. Moreover, in UCP group there were significant differences related to MACS levels, both for MA_NDH_ and AI.

None of the random variables (i.e. age, sex, side, presence/absence of mirror movements) showed significant interaction with MA_NDH_ and AI.

**Conclusions:**

These results confirm that actigraphy could provide, in a standardized setting, a quantitative description of differences between upper limbs activity.

**Trial registration:**

ClincalTrials.gov, NCT03054441. Registered 15 February 2017.

## Background

Cerebral palsy (CP), the most common cause of chronic childhood physical disability in industrialised societies, interests 2–3/1000 live births, up to 40–100/1000 among very premature and very low birth-weight infants [1]. Unilateral Cerebral Palsy (UCP, motor impairment on one side), constitutes the most frequent form of CP, comprising 30–40% of CP children [2, 3]. In children with UCP, presence of abnormal movement patterns of impaired UL is associated with lower levels of unimanual capacity and bimanual performance, impeding routine activities [4].

Clinicians interested in evaluation and treatment of ULs in children with UCP can choose from a wide range of assessment tools and classification systems. A systematic review of existing clinical tools [5] concludes that the best measure of unimanual capacity is the Melbourne Assessment of Unilateral Upper Limb Function (MUUL, upgraded to MA2) [6]; although the Shriners Hospital Upper Extremity Evaluation (SHUEE) [7], and Quality of Upper Extremity Skills Test (QUEST) [8] can also be utilised. The best performance-based measure of bimanual ULs activity in children with UCP was ascribed to the Assisting Hand Assessment (AHA) [9]. Finally, ABILHAND-Kids [10], a parent-report performance-based questionnaire, was also recommended.

Recently, thanks to progresses of Information and Communication technology (ICT), the use of technological solutions increases and, in parallel with traditional clinical assessments, additional information are provided [11], such as quantitative data during motor activity and asymmetries in the circadian motor activities [12, 13].

Also available devices on the market are increasing, such as wearable sensors (e.g. actigraphs, smartwatches or products or research devices such as ActiGraph, ActiWatch, GENEactiv, ETHOS). These types of sensors are commonly employed for the study of unbalanced conditions, such as handedness assessment in healthy [14] or unhealthy subjects [15] (e.g. upper limb mobility of post-stroke patients) by placing them symmetrically, i.e. on both wrists [11].

Technology measures provide quantitative data, however they are often designed for “standardized applications”, which means that the quantity and quality of data may be limited to the methodology of use. An advantage of wearable sensors is that the subject is free to behave in a more natural and spontaneous way.

The primary purpose of this study was to examine validity of ActiGraph GT3X+ worn on both wrists, to measure asymmetry in ULs use during AHA in subjects aged 3–25 years with UCP compared to age-matched TD. The hypothesis to be tested is that comprehensive indexes, as *activity counts*, derived from 3D accelerometric readings, can discriminate differences in the use of ULs in UCPs compared to TDs and detect asymmetry among individuals of UCP group related to clinical outcome measures (Assisting Hand Assessment scores and Manual Ability Classification System) in a standardized setting.

## Methods

Data were collected at IRCCS Fondazione Stella Maris, (FSM, Pisa, Italy) and at Queensland Cerebral Palsy and Rehabilitation Research Centre (QCPRRC, Brisbane, Australia). Potential participants were identified from a database of hemiplegic children at the Department of Developmental Neuroscience of FSM for children with UCP and from a clinical register at the QCPRRC. A convenience sample of TD volunteers were identified.

Eligible subjects were then invited to participate in the trial and informed consent was obtained from participants and/or parents prior to the beginning of assessment. Ethics approval was obtained from the ethics committees of participating hospitals and universities, specifically from the Tuscany Paediatric Ethics Committee (78/2016) in Italy and from the Child Health Queensland Ethics in Human Research as part of the PREDICT study (NHMRC 465128) in Australia. This study has been registered at the www.clinicaltrials.gov (NCT03054441).

Selection of UCP group was based on the following inclusion criteria:

i. confirmed diagnosis of UCP;
ii. age between 3 and 25 years;
iii. located in Italy or Queensland (Australia).

Exclusion criteria were:

i. medical complications that would interfere with study participation (e.g., uncontrolled seizures, epilepsy);
ii. predominantly dystonia or athetoid movement patterns;
iii. insufficient cognitive level to follow instructions;
iv. other progressive neurological disorders;
v. marked visual or hearing impairment.

Selection of healthy group was based on the following inclusion criteria:

i. age between 3 and 25 years;
ii. no clinically documented disorders;
iii. right-hand dominance;
iv. located in Italy or Queensland (Australia).

### Actigraphy

Each participant wore an activity monitor (wGT3X-BT Monitor, ActiGraph, Florida, FL, model 7164; 4.6 cm × 3.3 cm × 1.5 cm, 19 g) on each wrist. The devices were fastened to the wrist using custom-made Velcro wristbands. The ActiGraph GT3X+ monitor was selected for this study as it has been identified as a reliable instrument for measuring movement intensity [16, 17]. The wrist was identified as potentially viable location for wearing the ActiGraph GT3X+ monitor when assessing UL use in children with UCP.

### Assisting hand assessment (AHA)

The AHA (β-version 5.0) measures and describes effectiveness with which children with unilateral disability use the affected hand during bimanual activities [9]. AHA is scored from video recordings of semi-structured play activity, subsequently scored on the basis on 20 predefined items using a 4-point rating scale. There are different versions of AHA, analogous each other, allowing comparisons amongst different ages and detection of potential changes over time in the same individual [18]. UCP and TD participants undertook different versions of AHA assessment, depending on their age and cognitive level.

### Edinburgh handedness inventory (EHI)

The EHI is a simple quantitative method of assessing handedness, composed of 10 items (e.g. writing, drawing, throwing, using scissors) [19]. Handedness is calculated based on activities mainly done with right or left hand: it is the ratio between the difference of the two values divided by their sum, expressed as a percentage. It can range from − 100 (left-handed) to + 100 (right-handed). Participants of TD group were tested with EHI.

### Manual ability classification system (MACS)

The MACS classifies how children with CP use their hands to handle everyday objects. MACS describes five levels based on self-initiated ability to handle objects and need for assistance or adaption to perform manual routine activities. It is suitable for children between 4 and 18 years [20] and it has been adopted with young adults with UCP too [21–23].

### Setting

This trial was conducted in clinical environment during a play session. Each child wore two ActiGraph GT3X+ on both wrists, previously initialized and attached to Velcro-strap bracelets, during performance of age-appropriate AHA tests: Kids-AHA for children between 18 months and 12 years (free play for children younger than 5, alien game or fortress game for children aged between 5 and 12) or Ad-AHA board game “Go with the Ice Floe” for adolescents aged 12 or more.

AHA assessment was video recorded and the start and end times of the test were registered. In addition, MACS levels of UCP participants were rated by therapists together with participant’s family. EHI was performed as a structured interview to determine handedness/laterality of each TD participant and was scored using the online software http://zhanglab.wdfiles.com/local%2D%2Dfiles/survey/handedness.html. AHA was scored by a certified AHA rater from the video recording and expressed in AHA units.

Presence or absence of Mirror Movements in the DH hand during voluntary unimanual movements of the NDH and viceversa were evaluated in all the enrolled children.

### Actigraphic data

Data were recorded in 3 axes at 80 Hz (stored locally in the device) and downloaded using ActiLife v.6.13.3 software (ActiGraph, Pensacola, FL) to 1 Hz and converted to *activity counts* within the ActiLife v.6.13.3 software. Activity counts provide an index of intensity of physical activity at a precise time point: the higher the counts, the greater the intensity [24]. In addition, *activity counts* across three axes were combined using a Vector Magnitude.

Movement of each UL during AHA was quantified by mean activity count. Mean activity counts were defined as the mean of *activity counts* per second over the entire monitoring period. Mean Activity was extracted separately for the dominant (MA_DH_) and non-dominant hand (MA_NDH_), regarding values of Vector Magnitude. To quantify dominant hand movement relative to non-dominant hand movement during AHA, an asymmetry index was computed. Asymmetry index (AI) was calculated processing mean activity of each UL obtained from actigraphic data of the AHA collection following the Edinburgh Inventory formula:

$$ AI=\frac{MA_{DH}-{MA}_{NDH}}{MA_{DH}+{MA}_{NDH}}\ast 100 $$

An AI value of 0 indicates that both ULs contributed equally to activity during AHA assessment, while positive or negative values indicate greater contributions from either dominant or non-dominant UL compared to contralateral limb, respectively.

### Statistical analysis

All data were analyzed using the Statistical Package for Social Sciences (SPSS, version 20.0).

Two different linear mixed models were used to evaluate how dependent actigraphic variables (i.e. MA_NDH_ and AI) varied by group (TD vs UCP) and among MACS levels. In the model relative to the MA_NDH_, the group (TD vs UCP), the MACS levels, MA_DH_ and their interaction were entered as fixed effects. In the model relative to the AI values, the group (TD vs UCP) and the MACS levels and their interactions were considered as fixed effects. In both models age, sex, side of hemiplegia, presence/absence of mirror movements were specified as random effects, in order to correct estimates. Bonferroni corrections were carried out for multiple comparisons.

## Results

106 children (55 UCP and 51 TD) were evaluated and 100 met inclusion criteria.

Exclusion criteria were:

i. Movement disorder (*n* = 2);
ii. Insufficient cognitive levels (*n* = 3);
iii. Left-hand dominance for a TD subject (*n* = 1).

**Fig. 1:**
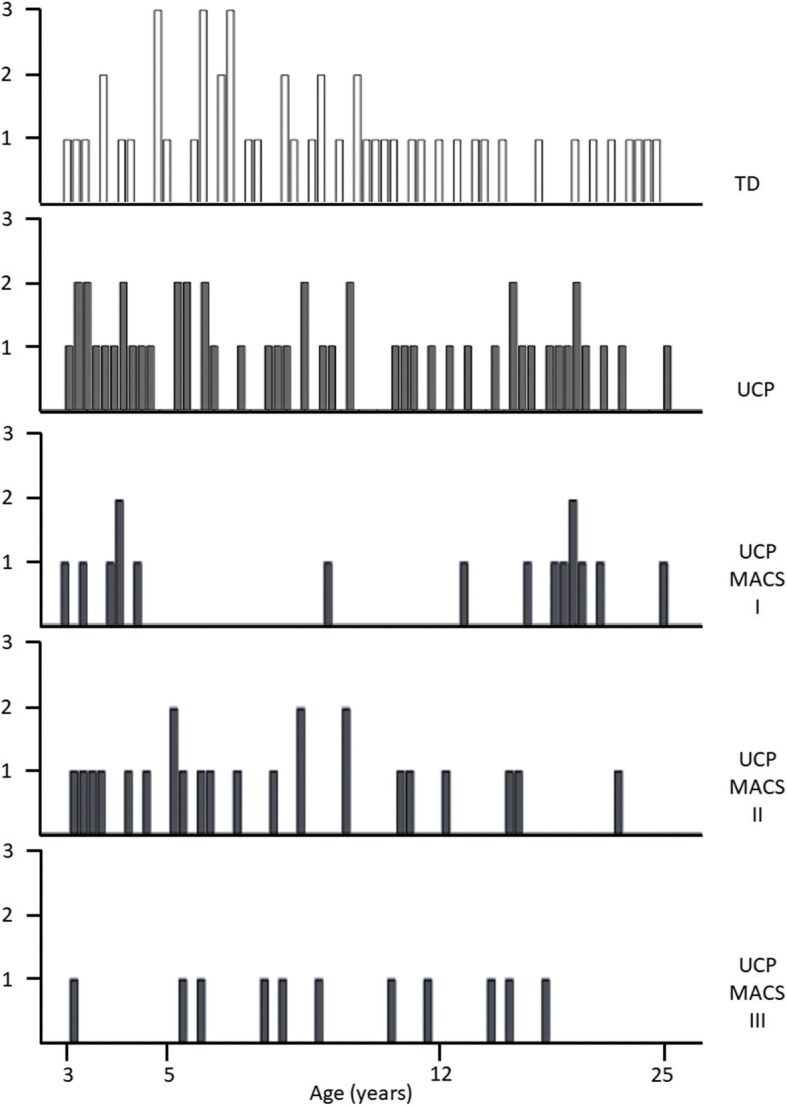
Counts per age of participants

The UCP group consisted of 50 subjects (mean age 9.93 ± 5.23 years, median 8.90, IQR 8.86, range 3–25). Gender and affected side were: male = 30, female = 20; right affected side = 33 and left = 17. 48 were Italian and 2 Australian. Children were classified as MACS level I = 16, MACS II = 23 and MACS III = 11^20^ (Fig. 1).

The TD group consisted of 50 subjects (mean age 10.14 ± 5.19 years, median 8.90, IQR 5.08, range 3–24.91, 30 Male, 20 Female). All TD presented right-hand handedness, as confirmed by EHI scores (> 0.8). 47 were Italian and 3 Australian.

**Fig. 2:**
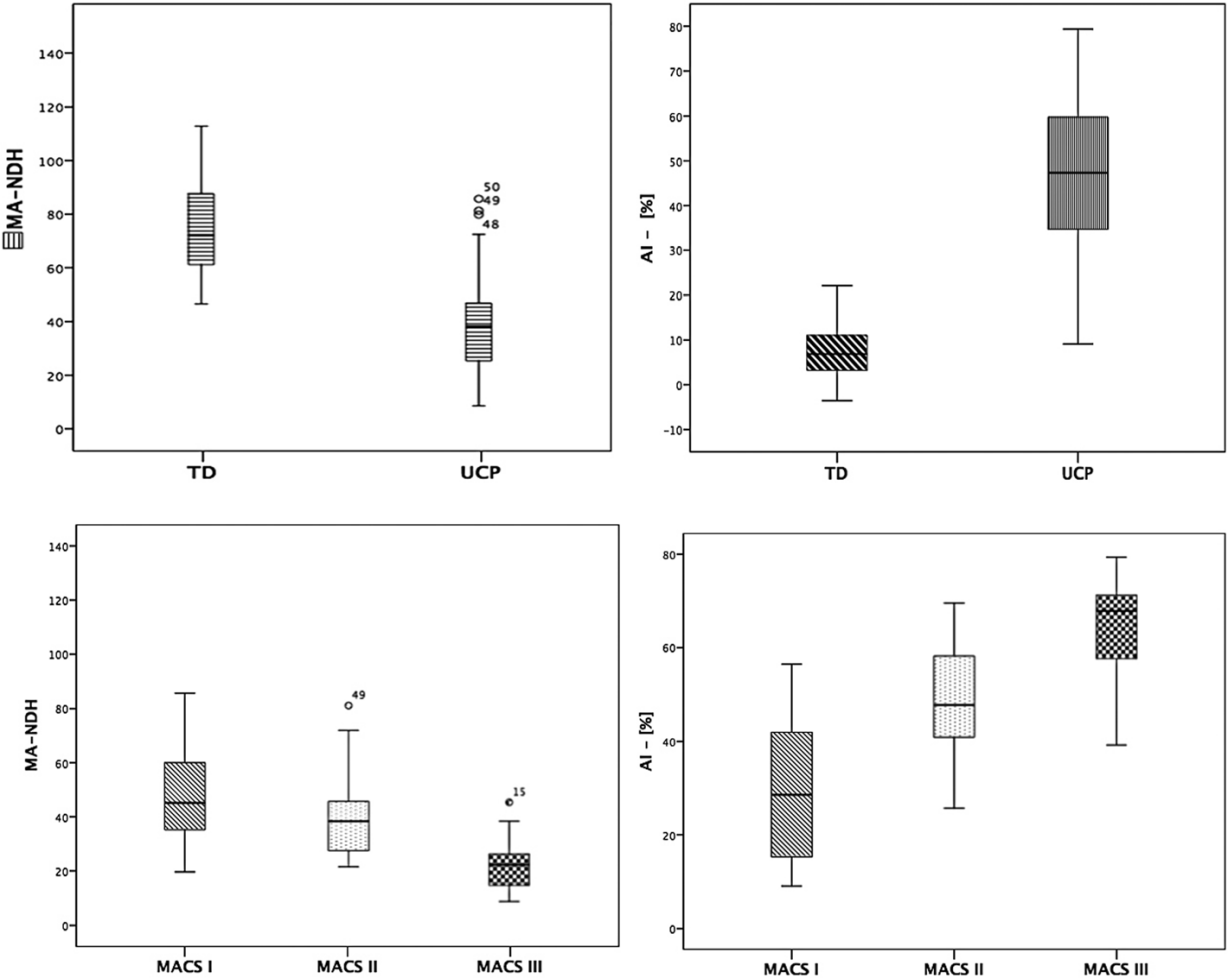
Mean activity of Dominant Hand (DH, upper-left quarter), Asymmetry Index (AI, upper-right quarter) in TD and UCP groups; Mean activity of Non Dominant Hand (NDH, lower-left quarter), Asymmetry Index (AI, lower-right quarter) of children with UCP grouped by MACS levels

Mean and Estimated mean values of MA_NDH_ and of AI for each group (UCP and TD) and for each MACS level are summarized in Tables 1 and 2. Boxplots are showed in Fig. 2.

**Table 1:** MA_NDH_ values and Estimated Mean Values of group (TD vs UCP) by MACS levels

		Mean	SD	Estimated Mean	SE	CI		p^*^	AIC^*^	BIC^*^
TD (*n* = 50)		74.712	16.239	78.537	1.888	74.788	82.286	< 0.001	806.511	809.064
UCP	Total (n = 50)	38.800	18.106	36.451	1.875	32.729	40.173	> 0.05		
	MACS I (*n* = 16)	48.335	19.248	49.374	3.303	42.817	55.930	< 0.001		
	MACS II (*n* = 23)	39.904	14.915	36.820	2.497	31.863	41.777	< 0.01		
	MACS III (*n* = 11)	22.623	11.184	23.160	3.720	15.774	30.545	-^**^		

*TD* Typically developing, *UCP* Unilateral Cerebral Palsy, *SD* Standard Deviation, *SE* Standard Error, *AIC* Akaike Information Criterion, *BIC* Bayesan Information Criterion

^*^p values related to the fixed model

^**^p values non available because there was no space in a single model to estimate them, due to few values present at MACS III level

**Table 2:** AI values and Estimated Mean Values of group (TD vs UCP) by MACS levels

		Mean	SD	Estimated Mean	SE	CI		p^*^	AIC^*^	BIC^*^
TD (*n* = 50)		7.266	6.309	7.266	1.468	4.355	10.177	< 0.001	735.737	738.301
UCP	Total (n = 50)	45.880	18.052	47.278	1.875	44.234	50.323	< 0.001		
	MACS I (n = 16)	29.889	16.374	29.888	2.593	24,742	35.034	< 0.001		
	MACS II (n = 23)	48.697	11.209	48.697	2.162	44.405	52.898	< 0.001		
	MACS III (n = 11)	63.250	12.603	63.250	3.127	57.043	69.457	-^**^		

*TD* Typically developing, *UCP* Unilateral Cerebral Palsy, *SD* Standard Deviation, *SE* Standard Error, *AIC* Akaike Information Criterion, *BIC* Bayesan Information Criterion

^*^p values related to the fixed model

^**^p values non available because there was no space in a single model to estimate them, due to few values present at MACS III level

Interactions among groups (UCP and TD) and among MACS levels in UCP group were significant both for MA_NDH_ (F_(2,95)_ = 127.437, *p* < 0.0001 and F_(2,95)_ = 13.904, *p* < 0.0001, respectively) and also for AI (F_(1,96)_ = 355.483, *p* < 0.0001 and F_(2,96)_ = 35.301, *p* < 0.0001, respectively). The UCP group showed significantly (p < 0.0001) lower MA_NDH_ values than TD and also comparisons among MACS were significant (MACS I vs MACS II, *p* < 0.05, and MACS I vs MACS III *p* < 0.001; MACS II vs MACS III, *p* < 0.05) with estimated MA_NDH_ values that decreased from the TD to the MACS I, II and III, respectively. However, only in the TD group the MA_NDH_ values were related to the MA_DH_, while it was not significant in the UCP one.

As concerning the AI, UCP group showed significantly higher values than TD (*p* < 0.001) with a increasing significant trend from the TD values to the MACS III and significant differences among MACS (MACS I vs MACS II, *p* < 0.001, and MACS I vs MACS III *p* < 0.001; MACS II vs MACS III, *p* = 0.001).

Variables as age, sex, side of hemiplegia, presence/absence of mirror movements did not show significant interaction with MA_NDH_ and AI. We included these variables in the models as random factors, in order to get estimates corrections.

## Discussion

To our knowledge, this is the first study aimed specifically to evaluate the validity of ActiGraph GT3X+ for detecting asymmetry in UL use in subjects with UCP. It explores and reveals significant differences in bimanual activities between TD and UCP and among different UCP levels.

By systematically analyzing the literature in this field [25], we found few studies on the asymmetric use of one limb in a population of children with UCP. Lundh and collaborators [26] assessed the degree of deviation and asymmetry in upper and lower extremities during walking, and Coker-Bolt and collaborators [27] determined the feasibility of the use of actigraphy before, during and after a CIMT program.

In the present study the semi-structured setting of AHA and the high acceptance of ActiGraphs (similar to traditional digital watch) allowed the detection of the spontaneous use of Uls (mean activity) in both TDs and UCPs. The mean activity of the upper limb on both wrists relates not only to the quantity of movement in terms of their, but also to the general concept of amount, that is the whole measurement of activity, including also its intensity [28]. ActiGraphs were able to quantify differences in mean activity of the NDH, with DH as covariate, showing that there were significant differences between the two groups (TC Vs UCP) while for single group effect the MA_NDH_ was significant only in the TD. Moreover, for TDs, AI was very low, demonstrating a high cooperation of between the two hands. This is in direct contrast to the significantly higher asymmetry values in UCPs, since manual activities typically require co-operation between both hands which also tend to be specialized for different functions [29] e.g. when unscrewing a jar lid or buttoning a shirt, one hand (typically the non dominant) holds the object while the other (typically the dominant) acts upon it. NDH therefore has the role of stabilizing objects, providing in the meantime a spatial reference in which DH manipulates. Stabilization performed by NDH is not always related to a fixed position, in fact this stabilization could be static (immobile position of NDH) or dynamic (several re-grasping and readjusting of NDH grip). Dynamic stabilization is, for instance, performed during handwriting, when NDH periodically repositions the paper to guarantee the right orientation for DH. In the present work, we did not analyse each single task separately, but we carried out a global analysis during a session of semi-structured playing activity, as the AHA session: in fact, all objects are presented without specific instructions, but observing the spontaneous behaviour of the child. Due to the variability of adopted strategies for accomplish the task, it does not make sense to separate each task. Our hypothesis, and our finding, is that actigraphy allows to measure the activity of upper limbs in a frame of natural playing activity and not in a standardized and defined activity. Furthermore, there are already in literature some papers demonstrating the reliability and validity of actigraphs in measuring physical activity across a wide variety of activities [28] and one of the perspectives of this work is in fact to measure physical activity intensity in natural environments to document it in real-world activities.

An interesting finding was the lack of relationship in children with UCP between the NDH and DH values. It could be related to the findings of other studies [30–32], where it is reported that the nonparetic hand of hemiplegic children was significantly impaired, although to a lesser degree than the paretic hand of children with UCP. The three outliers (#48, #49 and #50) with higher MA_NDH_ values were the same subjects with higher AHA values.

Another important finding was the differences in actigraphic data according to MACS levels. MACS classifies how CP children use their hands to manipulate objects in routine activities TD and all three MACS classes (I, II and III) in UCP have significantly different MANDH vs MADH values [29]. When comparing MACS levels with AI, the data confirmed the significant differences among levels with a significant increase in AI. In fact, children with UCP at MACS level III have the highest level of UL asymmetry.

The lack of age effects in all the models seems to be in contrast with the recent literature that shows age influences on the AHA scores, with a less effective spontaneous use of the NDH in bimanual tasks [33, 34]. However, the age effect has been explored in longitudinal studies, evaluating the individual trend. In the present paper, the data across ages are acquired from different patients.

Taking account all the results, we can summarize that the use of actigraphs, during the AHA assessment, allowed us to obtain a quantitative and objective measure of the different use of NDH and DH and the relative asymmetry. Moreover, the statistical models allowed us to discriminate the TD from the UCP and also in the UCP among different severity MACS levels.

### Limitations of the study

The main limitation point is that the rotations at a relative slow angular speed of the actigraph may result in an increase of the *activity counts* due to the changes in the projection of the vertical gravity acceleration, without any meaningful linear accelerations. Therefore, the use of other types of sensors, like accelerometer combined with gyroscopes, would be interesting, for a less biased assessment of level-of activity. Moreover, we did not find any effects on the side of hemiplegia in the analysed models. However, another interesting side aspect, which could be analysed in future studies, is the difference between the attentional and perceptual problems in left Vs. right hemiplegia, as shown in Katz et al. [35] and Sterzi et al. [36]. These aspects can influence the spatial orientation with a consequent impact also on manual function.

## Conclusions

This study confirmed that actigraphs are valid tool, able to measure the amount of movement of ULs to quantify comparisons between limbs and confirmed construct validity between actigraphic data and MACS levels and severity of bimanual coordination on AHA, without requiring excessive time or effort.

The clinical implications are that quantitative data give a more detailed description of UL activity and symmetry, with the opportunity of having a sensitive tool for detecting spontaneous and intervention-induced changes [37, 38].

In fact, results of standardized clinical scales are often dependent on the experience of the therapist and although the AHA requires a certificated training course for operators, the score is highly related to the scorer; on the contrary, the actigraphic assessment is based on objective acquisition. In this work, we showed that the AHA setting is suitable for measuring asymmetries in children during a semi-structured playing session. Moreover, actigraphs can be used to explore hand activity in natural situation, at home, and not in the clinical setting. Future perspectives may confirm that actigraphy could become a reliable and non-invasive tool for systematically measuring UL activity, addressing further studies of bimanual activities and handedness in clinical situations. Actigraphy could become a “holter” of motor behavior of upper limb activity, not only for discriminating deviating use of the limb but for longitudinally assessing and monitoring the upper limb asymmetries and for detecting changes in manual daily life activities during and after rehabilitation treatments [38].

## Abbreviations

Ad: Adolescents
AHA: Assisting Hand Assessment
AI: Asymmetry Index
CI: Confidence Intervals
CP: Cerebral Palsy
DH: Dominant Hand
EHI: Edinburgh Handedness Inventory
MACS: Manual Ability Classification System
MA_DH_: Mean Activity of Dominant Hand
MA_NDH_: Mean Activity of Non-dominant Hand
NDH: Non-dominant Hand
TD: Typically developing
UCP: Unilateral Cerebral Palsy
UL: Upper Limb
